# Characterization of an antibody that recognizes peptides containing D-β-aspartyl residues

**Published:** 2012-04-21

**Authors:** Kenzo Aki, Norihiko Fujii, Takeshi Saito, Noriko Fujii

**Affiliations:** Research Reactor Institute, Kyoto University, Kumatori, Osaka, Japan

## Abstract

**Purpose:**

Biologically uncommon D-β-aspartyl (D-β-Asp) residues have been detected in proteins from various human tissues from elderly donors and are connected with cataract, age-related macular degeneration, Alzheimer disease and UV-irradiated skin. In a previous study, we prepared a highly specific antibody against the peptide Gly-Leu-D-β-Asp-Ala-Thr-Gly-Leu-D-β-Asp-Ala-Thr-Gly-Leu-D-β-Asp-Ala-Thr (designated peptide 3R) that corresponds to three repeats of positions 149–153 of human lens αA-crystallin. This antibody clearly distinguishes between the different configurations of the Asp residue in that it reacted strongly with the D-β-Asp-containing peptides but did not react with L-α-Asp-, L-β-Asp-, or D-α-Asp-containing peptides. However, it remains unclear whether the antibody recognizes the amino acid sequences surrounding the D-β-Asp residue. The purpose of the present study is to elucidate the sequence dependency of the epitope of the antigen.

**Methods:**

To clarify the properties of the anti-peptide 3R antibody, we used F-moc (9-fluorenylmethoxycarbonyl) solid phase chemistry to synthesize various peptides and analogs based on the peptides T18 (I^146^QTGLDATHAER^157^) and T6 (T^55^VLDAGISEVR^65^) which correspond to amino acid sequences 146–157 and 55–65, respectively of human αA-crystallin. The specificity of antibody was confirmed by ELISA (enzyme-linked immunosorbent assay) using these peptides.

**Results:**

The anti peptide 3R antibody specifically recognized D-β-Asp residues and does not react with other configurations of Asp such as the L-α, L-β, D-α isomers in peptides. When the Ala in the peptide was replaced by other amino acid residues, the antibody did not react with the antigen. The antibody requires the sequence Leu-D-β-Asp-Ala to detect D-β-Asp containing proteins in living tissue.

**Conclusions:**

The anti peptide 3R antibody is a powerful and easy tool for detection of D-β-Asp containing proteins in living tissues from patients with age-related diseases. However, to detect the D-β-Asp containing proteins in the living tissues using the anti-peptide 3R antibody, the protein must contain the sequence Leu-D-β-Asp-Ala.

## Introduction

Proteins consist exclusively of L-amino acids in living tissues. However, D-aspartyl (Asp) residues have been detected in various proteins of tooth [1], eye lens [2-5], aorta [6], brain [7,8], bone [9], and skin [10,11] in elderly donors. Importantly, the proteins containing D-amino acids are derived from tissues that are metabolically inert. Thus, D-amino acid residues arise due to racemization of amino acids in the protein during the life span of the individual. Of all the naturally occurring amino acids, aspartic acid (Asp) is the most susceptible to racemization. However, the specific sites in which racemization of Asp residues in proteins occurs has not been determined except for lens and brain proteins in the reports described above.

In our previous study, we found that specific Asp residues in αA-crystallin (Asp 58 and Asp 151) [3], αB-crystallin (Asp 36 and Asp 62) [4], and βB2-crsytallin (Asp 4) [5] in the human lens were highly inverted from the L-isomer to the D-isomer and the peptide bond isomerized from the normal α-linkage to a β-linkage. In proteins these isomers can cause major changes in structure, since different side chain orientations can induce an abnormal peptide backbone. Therefore, the presence of the isomers may be one of triggers of abnormal aggregation and can induce the partial unfolding of protein leading to a disease state. In fact, the previous study clearly showed that α-crystallin containing large amounts of D-β-Asp undergoes abnormal aggregation to form massive and heterogeneous aggregates, leading to loss of its chaperone activity [12]. Similarly, we observed the accumulation of D-β-Asp containing proteins in sun-damaged face skin from elderly people [11]. The abnormal protein was localized to the elastic fiber-like structures of dermis from elderly donors with actinic elastosis [13].

These findings indicate that D-β-Asp residues are present widely and arise due to racemization of amino acids in various proteins during the lifespan of the individual. Therefore, it is necessary to be able to detect D-β-Asp containing proteins in the living tissues of elderly donors. We have detected specific sites of D-β-Asp in proteins from cataractous lenses using the following steps: 1) Purification of the target protein using various column chromatographic methods, 2) Digestion of the protein obtained in step 1 with trypsin, 3) Separation of the tryptic peptides obtained in step 2 by reversed phase high performance liquid chromatography (RP-HPLC), 4) Identification of the tryptic peptides by sequence analysis and mass spectrometry, 5) Analysis of β/α ratio of Asp in peptide ; since the β-Asp containing peptides are clearly separated from normal α-Asp containing peptides upon RP-HPLC, the β/α ratios of the Asp residues in the peptides are calculated from the ratio of the peak areas. The confirmation of a β-Asp residue in peptides is performed by protein sequencing because the β-Asp containing peptides are resistant to Edman degradation. 6) Analysis of D/L ratio of Asp in peptide; to determine the D/L ratio of the Asp residue in a peptide, the identified tryptic peptides are hydrolyzed and derivatized to form diastereoisomers using *o*-phthalaldehyde (OPA) and *N*-tert-butyloxycarbonyl-L-cysteine (Boc-L-cys). The diastereoisomers are then subjected to RP-HPLC and the D/L ratio of amino acids determined by analyzing the ratio of the respective peak areas. According to the above methods, we determined that the D/L ratios of Asp 58 in the T6 peptide (TVLD^58^SGISEVR), Asp 151 in the T18 peptide (IQTGLD^151^ATHAER) of αA-crystallin and Asp 4 in the T1 peptide (MASD^4^HQTQAGK) of βB2-crystallin from elderly donors were 3.2, 5.8 and 3.2, respectively. This method is precise and quantitative for the analysis of the contents of specific D-β-Asp sites in protein. However, it is time consuming to detect D-β-Asp in any protein because of the complicated methodology which includes several column steps. For this reason, we prepared a highly specific antibody against the peptide Gly-Leu-D-β-Asp-Ala-Thr-Gly-Leu-D-β-Asp-Ala-Thr-Gly-Leu-D-β-Asp-Ala-Thr (designated peptide 3R) that corresponds to three repeats of positions 149–153 of human lens αA-crystallin. This antibody clearly distinguishes the configuration of the Asp residue, in that it reacts strongly with D-β-Asp-containing peptides but does not react with L-α-Asp-, L-β-Asp-, or D-α-Asp-containing peptides [14]. Immunohistochemical staining of human lens with this antibody demonstrated that D-β-Asp-151-containing αA-crystallin was predominantly localized to the core of aged human lens while in the young lens, immunostaining was not observed [14]. The result was consistent with that of our previous biochemical study [3]. Therefore, peptide 3R immunoreactive protein is considered to be equivalent to D-β-Asp-containing peptide/protein. Using this antibody, D-β-Asp-containing peptide/proteins were observed in nonpigmented ciliary epithelial cells, in drusen, and in the sclera [15] of eye and in the sun-damaged skin of elderly donors [11] and epithelial skin of mouse [16]. Moreover, the previous study reported that D-β-Asp-containing peptide/protein and advanced glycation end products (AGEs), which are induced by a nonenzymatic glycation under oxidative stress, commonly accumulated in the same proteins during aging [16]. This result means that the isomerization of Asp and AGE are the results of oxidative stress. Therefore, rapid and easy analysis of the D-β-Asp-containing peptide/proteins is required. The purpose of the present study was to investigate the general applicability of the antibody and the sequence dependency of the epitope of the antigen.

## Methods

### The synthesis of the peptides containing four different Asp isomers

We synthesized T18 peptides (IQTGLDATHAER) and T6 peptides (TVLDSGISEVR) of αA-crystallin and T1 peptide of βB2-crystallin (MASDHQTQAGK) together with related peptides in which amino acids surrounding the Asp residues were substituted. In addition to the above peptides, we synthesized a peptide (NRKDAEEWFNQK) from keratin 10 of mouse skin. Furthermore, isomeric peptides containing 4 different Asp isomers, L-α-Asp-, L-β-Asp-, D-α-Asp or D-β-Asp were also synthesized. The peptides synthesized in this study are shown in Table 1.

**Table 1:** A list of the synthetic peptides used for the experiment.

**Group**	**Sequence**	**Peptide name**	**Remarks column**
Leu-Asp-Ala	IQTGLD(Lα)ATHAER	αA-crystallin T18 Lα	→Figure 2
	IQTGLD(Lβ)ATHAER	αA-crystallin T18 Lβ	
	IQTGLD(Dα)ATHAER	αA-crystallin T18 Dα	
	IQTGLD(Dβ)ATHAER	αA-crystallin T18 Dβ	
	TVLD(Lα)AGISEVR	αA-crystallin T6 SA Lα	→Figure 4
	TVLD(Lβ)AGISEVR	αA-crystallin T6 SA Lβ	
	TVLD(Dα)AGISEVR	αA-crystallin T6 SA Dα	
	TVLD(Dβ)AGISEVR	αA-crystallin T6 SA Dβ	
Leu-Asp-Y	TVLD(Lα)SGISEVR	αA-crystallin T6 Lα	→Figure 3
	TVLD(Lβ)SGISEVR	αA-crystallin T6 Lβ	
	TVLD(Dα)SGISEVR	αA-crystallin T6 Dα	
	TVLD(Dβ)SGISEVR	αA-crystallin T6 Dβ	
	TVLD(Lα)RGISEVR	αA-crystallin T6 SR Lα	→Figure 5
	TVLD(Lβ)RGISEVR	αA-crystallin T6 SR Lβ	
	TVLD(Dα)RGISEVR	αA-crystallin T6 SR Dα	
	TVLD(Dβ)RGISEVR	αA-crystallin T6 SR Dβ	
X-Asp-Y	MASD(Lα)HQTQAGK	βB2-crystallin T1Lα	→Figure 6
	MASD(Lβ)HQTQAGK	βB2-crystallin T1Lβ	
	MASD(Dα)HQTQAGK	βB2-crystallin T1Dα	
	MASD(Dβ)HQTQAGK	βB2-crystallin T1Dβ	
X-Asp-Ala	IQTGLD(Lα)ATHAER	αA-crystallin T18 LR Lα	→Figure 7
	IQTGLD(Dβ)ATHAER	αA-crystallin T18 LR Dβ	
	NRKD(Lα)AEEWFNQK	keratin 10 (mouse) 330–341Lα	→ Figure 8
	NRKD(Dβ)AEEWFNQK	keratin 10 (mouse) 330–341Dβ	

The peptides containing four different Asp isomers were synthesized by Fmoc (9-ﬂuorenylmethoxycarbonyl) solid-phase chemistry using an automated solid-phase peptide synthesizer (PSSM-8; Shimadzu, Kyoto, Japan). Fmoc-L-Asp (OtBu)-OH, Fmoc-D-Asp (OtBu)-OH, Fmoc-L-Asp-OtBu and Fmoc-D-Asp-OtBu were used as building blocks to synthesize L-α-, D-α-, L-β- and D-β-isomers, respectively. The coupling reaction was performed using each Fmoc amino acid (10 equiv), PyBOP (10 equiv), 1-hydroxybenzotriazole (HOBt) (10 equiv), *N*-methylmorpholine (7.5 equiv) in dimethylformamide (DMF). The N-terminal Fmoc group was de-blocked with 20% piperidine in DMF. Simultaneous cleavage of the peptide from the resin and removal of the protective groups was achieved by treatment with a cocktail containing 90% TFA, 5% 1,2-ethanedithiol and 5% thioanisole.

### Purification of the synthetic peptides

The crude peptides were purified by RP-HPLC using a C18 column (Capcell pak C18 ACR, 30 × 250 mm; Shiseido, Tokyo, Japan) with a linear gradient of 0%–50% acetonitrile in the presence of 0.1% TFA, at a flow rate of 3.0 ml/min, with monitoring at 215 nm. The purity of each peptide was confirmed to be >95% by analytical RP-HPLC.

### Characterization of the synthetic peptides by MALDI-TOFMS

The purity of the synthetic peptides was confirmed by mass spectrometry. The desalted peptide solution (5 μl) was mixed with an equal volume (5 μl) of matrix solution. As matrix solution, α-cyano-4-hydroxycinnamic acid (CHCA, 10 mg) was dissolved in 1 ml of a solution containing a 1:1 ratio of aqueous 0.1% trifluroacetic acid and acetonitrile. The mixture of peptide and matrix was spotted onto a 384-spot MALDI target and then dried. All spectra were obtained using a matrix-assisted laser desorption/ionization time-of-flight (MALDI-TOF) mass spectrometer (AXIMA TOF2; Schimadzu, Kyoto, Japan). The MALDI-TOF equipment was operated with a nitrogen laser with a wavelength of 337 nm and ion acceleration voltage of 20 kV. The data were collected in reflection mode as signals of positive ions.

### Antibody against D-β-Asp containing peptide

The preparation and characterization of the antibody used are described in our previous paper [14]. Briefly, the polyclonal antibody against the peptide Gly-Leu-D-β-Asp-Ala-Thr-Gly-Leu-D-β-Asp-Ala-Thr-Gly-Leu-D-β-Asp-Ala-Thr (designated peptide 3R), containing three repeats of position 149–153 of human αA-crystallin optic isomer, was purified from rabbit serum by affinity chromatography using peptide 3R and bovine αA-crystallin as ligands. The anti peptide 3R antibody clearly distinguished the configuration of the Asp-residue, such that it reacted very strongly with D-β-Asp containing peptide but did not react with L-α-Asp, D-α-Asp and L-β-Asp-containing peptides [14]. The antibody clearly recognized the presence of D-β-Asp containing αA-crystallin in aged human lenses [14].

### Enzyme-linked immunosorbent assay (ELISA)

To confirm the reactiveness of the antibody, ELISAs were performed using ELISA Plates from IWAKI (Tokyo, Japan). Initially, target peptide was adjusted with PBS to 1 μg/ml, and 50 μl/well added to the ELISA plate. The plate was incubated over night at 4 °C. After incubating, wells were washed once with washing solution (0.1% PBS-Tween). After washing, 200 μl/well blocking solution (5% BSA-PBS) was added. The plate was then incubated for one hour at 25 °C. After incubating wells were washed 3 times with washing solution. After washing, 50 μl/well of antibody diluted 10 times and 100 times with PBS was added. The plate was incubated for one hour at 25 °C. After incubation, the wells were washed 5 times with wash solution. After washing, 50 μl/well of secondary antibody ((HRP)-conjugated anti rabbit IgG antibody) diluted 1:10,000 with PBS was added. The plate was incubated for one hour at 25 °C. After incubation wells were washed 5 times with wash solution. After washing 100 μl/well, stain solution (stabilized chromogen; Invitrogen, Carlsbad, CA) was added. The plate was incubated for 30 min at 25 °C. After incubation, 100 μl/well STOP solution (Invitrogen) was added. The absorbance of the wells was measured at 450 nm using a Wallac plate reader (PerkinElmer, Waltham, MA).

## Results and Discussion

**Figure 1:**
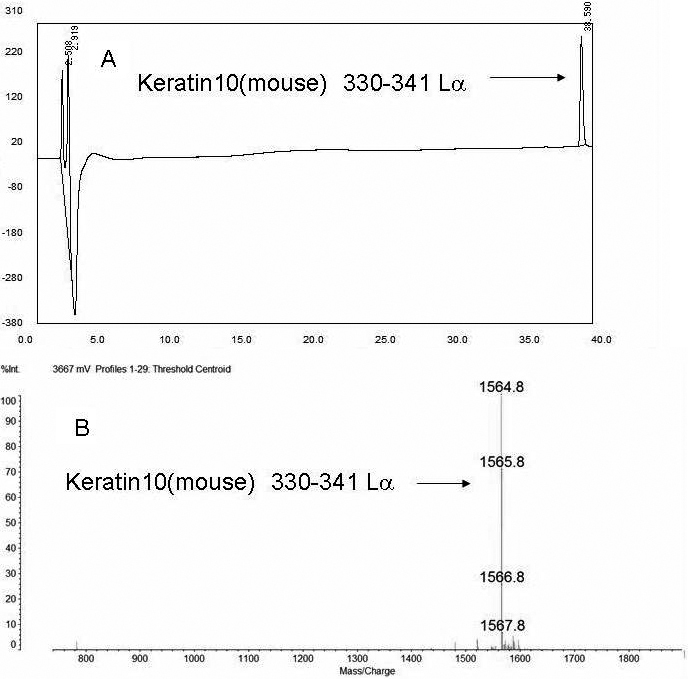
The purity of the synthesized peptides. **A**: A typical RP-HPLC elution profile of the purified keratin10 (330–341) peptide. **B**: Mass analysis of the purified keratin10 (330–341) peptide. The observed mass of the keratin10 (330–341) peptide is consistent with the theoretical mass. The purity of the peptide was confirmed to be more than 99%. The purities of all synthesized peptides were confirmed by a combination of RP-HPLC and mass analysis.

Figure 1 shows a typical RP-HPLC chromatogram of the keratin 10 peptide synthesized in this study. The purity of the peptides was confirmed by RP-HPLC and mass analysis.

Our previous chemical analysis clearly showed that Asp 151 in the T18 peptide and Asp 58 in the T6 peptide of αA-crystallin [3] and Asp 4 in the T1 peptide of βB2-crystallin [5] were highly inverted in the lens of elderly donors. In the previous study, we prepared a highly specific antibody against the peptide 3R (GLD(Dβ)ATGLD(Dβ)ATGLD(Dβ)AT) that corresponds to three repeats of a part of the T18 peptide of αA-crystallin. The antibody recognizes the Dβ-Asp residues but not the other Asp isomers such as Lα, Lβ and Dα residues in the T18 peptide of αA-crystallin. The antibody reacted with aged human αA-crystallin that contains an abundance of Dβ-Asp residues, while the antibody did not react with young human αA-crystallin that has very low levels of Dβ-Asp residues [14]. The results clearly indicate that the anti peptide 3R antibody recognized only Dβ-Asp residues in peptides and protein. However, it is unclear whether the reactivity of the antibody changes depending on the amino acid sequences surrounding the Dβ-Asp residues. Therefore, we synthesized the T18 and T6 peptides and related peptides, in which we replaced several amino acids surrounding the Asp residues. In addition to the above peptides, we also synthesized a peptide (NRKDAEEWFNQK) from keratin 10 protein of mouse skin. Furthermore, synthetic peptides containing 4 different Asp isomers, L-α-Asp-, L-β-Asp-, D-α-Asp or D-β-Asp were also prepared for all peptides. Using these peptides, we evaluated the properties of the anti-peptide 3R antibody.

**Figure 2:**
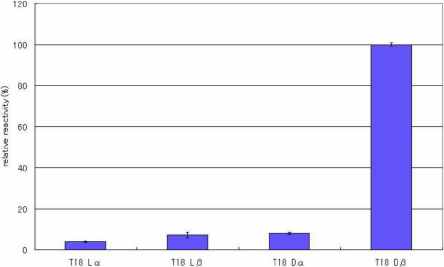
The reactivity of the peptide 3R antibody toward T18 peptide isomers. Results of ELISA assay for determining the specificity of the anti-peptide 3R antibody using 4 different αA-cystallin T18 isomeric peptides (IQTGLDATHAER) containing 4 Asp isomers, L-α-Asp-, L-β-Asp-, D-α-Asp and L-β-Asp. ELISAs were performed with a combination of a 100 times dilution for the first antibody and a 10,000 times dilution for the second antibody. The reactivity of the isomeric peptides can be expressed as a percentage using the reactivity toward T18 Dβ as 100%. (n=3, ±2σ [sigma]).

**Figure 3:**
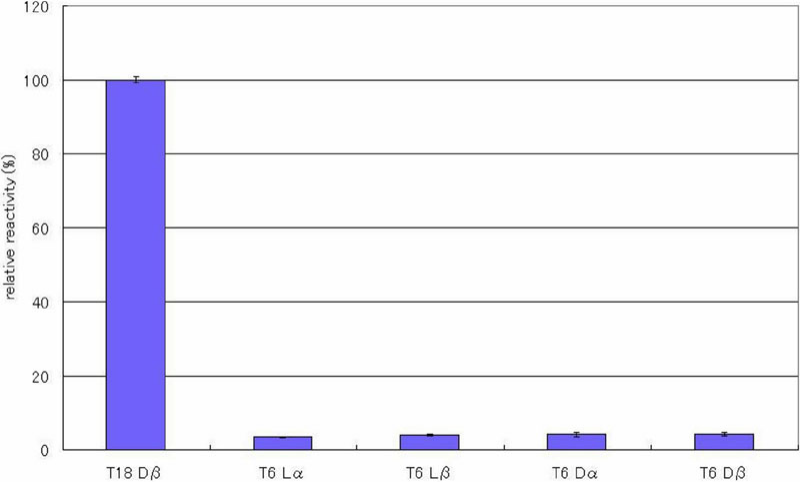
The reactivity of the peptide 3R antibody toward T6 peptide isomers. Results of ELISA assay for determining the specificity of the anti-peptide 3R antibody using 4 different αA-cystallin T6 isomeric peptides (TVLDSGISEVR), containing 4 Asp isomers, L-α-Asp-, L-β-Asp-, D-α-Asp and L-β-Asp. ELISAs were performed with a combination of a 100 times dilution for the first antibody and a 10,000 times dilution for the second antibody. The reactivity of the peptides can be expressed as a percentage using the reactivity toward T18 Dβ as 100%. (n=3, ±2σ [sigma]).

**Figure 4:**
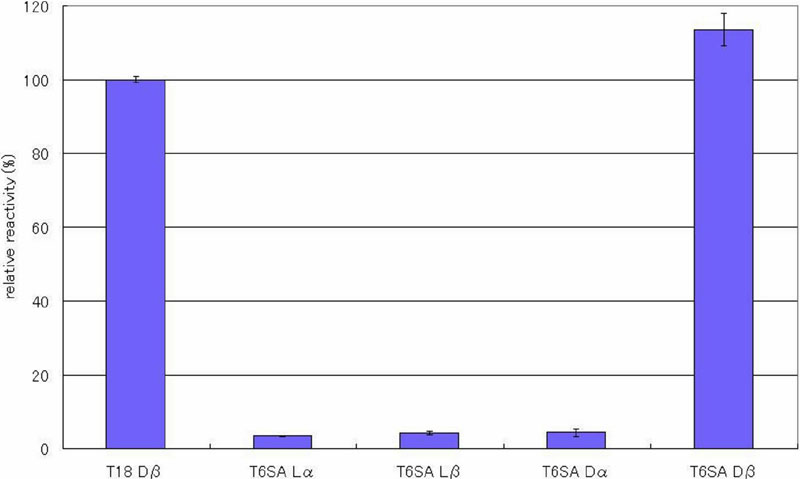
The reactivity of the peptide 3R antibody toward T6 SA peptide isomers. Results of ELISA assay for determining the specificity of the anti-peptide 3R antibody using αA-crystallin T6 SA peptides (TVLDAGISEVR), in which the Ser residue following the Asp residue was replaced by Ala, and in which the 4 different Asp isomers (L-α-Asp-, L-β-Asp-, D-α-Asp and L-β-Asp) were included. ELISAs were performed with a combination of a 100 times dilution for the first antibody and a 10,000 times dilution for the second antibody. The reactivity of the peptides can be expressed as a percentage using the reactivity toward T18 Dβ as 100%. (n=3, ±2σ [sigma]).

**Figure 5:**
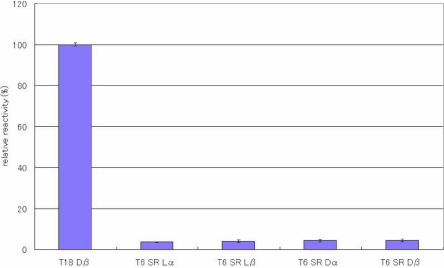
The reactivity of the peptide 3R antibody toward T6 SR peptide isomers. Results of ELISA assay for determining the specificity of the anti-peptide 3R antibody using T6 SR(TVLDRGISEVR), in which the Ser residue following the Asp residue was replaced with Arg, and in which the 4 different Asp isomers (L-α-Asp-, L-β-Asp-, D-α-Asp and L-β-Asp) were included. ELISAs were performed with a combination of a 100 times dilution for the first antibody and a 10,000 times dilution for the second antibody. The reactivity of the peptides can be expressed as a percentage using the reactivity toward T18 Dβ as 100%. (n=3, ±2σ [sigma]).

**Figure 6:**
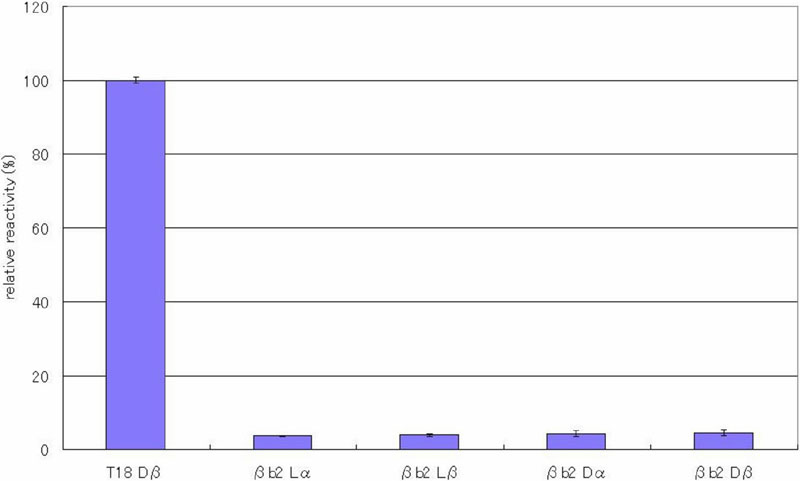
The reactivity of the peptide 3R antibody toward bB2 T1 peptide isomers. Results of ELISA assay for determining the specificity of the anti-peptide 3R antibody using βB2-cystllin T1 peptide (MASD^4^HQTQAGK) isomers, in which the 4 different Asp isomers (L-α-Asp-, L-β-Asp-, D-α-Asp and L-β-Asp) were included. ELISAs were performed with the combination of a 100 times dilution for the first antibody and a 10,000 times dilution for the second antibody. The reactivity of the peptides can be expressed as a percentage using the reactivity toward T18 Dβ as 100%. (n=3, ±2σ [sigma]).

**Figure 7:**
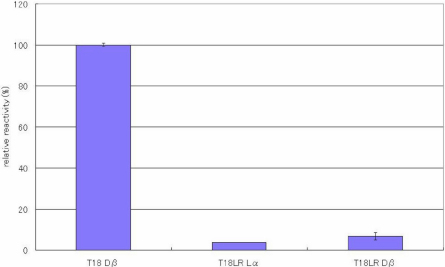
The reactivity of the peptide 3R antibody toward T18 LR peptide isomers. Results of ELISA assay for determining the specificity of the anti-peptide 3R antibody using T18LR of αΑ-cystallin (IQTGRDATHAER) in which the Leu residue before the Asp residue was replaced with Arg, and in which the 2 different Asp isomers (L-α-Asp- and L-β-Asp) were included. ELISAs were performed with a combination of a 100 times dilution for the first antibody and a 10,000 times dilution for the second antibody. The reactivity of the peptides can be expressed as a percentage using the reactivity toward T18 Dβ as 100%. (n=3, ±2σ [sigma]).

**Figure 8:**
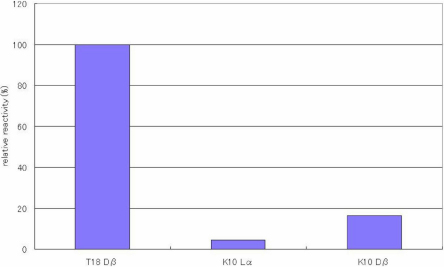
The reactivity of the peptide 3R antibody toward K10 peptide isomers’ Results of ELISA assay for determining the specificity of the anti-peptide 3R antibody using keratin 10 (residues 330–341 - NRKDAEEWFNQK), peptide isomers in which the 4 different Asp isomers were included. ELISAs were performed with a combination of a 100 times dilution for the first antibody and a 10,000 times dilution for the second antibody. The reactivity of the peptides can be expressed as a percentage using the reactivity toward T18 Dβ as 100%. (n=3, ±2σ [sigma]).

ELISAs were performed to evaluate the specificity of the antibody toward the configuration of Asp residues in the peptides. Figure 2, Figure 3, Figure 4, Figure 5, Figure 6, Figure 7, and Figure 8 shows the results of the ELISAs with the combination of the 100 times dilution for the first antibody and 10,000 times dilution for the second antibody. As shown in Figure 2, the ELISAs clearly demonstrate that the anti-peptide 3R antibody reacts strongly with the T18 peptide (IQTGLDATHAER), containing a D-β-Asp residue while the antibody scarcely reacted with T18 peptides containing the other isomers such as L-β-Asp, D-α-Asp, L-α-Asp. The reactivity of the isomeric peptides can be expressed as a percentage using the reactivity of T18 Dβ as 100%. Figure 2 clearly indicates that the anti-peptide 3R antibody is specific for the D-β-Asp residue of the T18 peptide. The result is completely consistent with that of our previous study [14]. In contrast, the D-β-Asp residue in the T6 peptide (TVLDSGISEVR) was not recognized at all by the anti-peptide 3R antibody. The reactivities of all isomeric T6 peptides were less than 10% that toward the D-β-Asp residue of the T18 peptide (Figure 3). Therefore, we synthesized T6 SA peptides (TVLDAGISEVR), in which the Ser residue after the Asp residue was replaced with Ala, and in which the 4 different Asp isomers were included. We then examined the reactivities of the peptides using the anti-peptide 3R antibody. The results clearly indicate that the antibody strongly recognizes the D-β-Asp residue of the T6SA peptide (TVLDAGISEVR) at a level 100% that of the reactivity toward the D-β-Asp residue of the T18 peptide, while the antibody barely reacts with the T6SA peptides (less than 10%) containing the L-β-Asp, D-α-Asp, L-α-Asp isomers (Figure 4). This result suggests that the anti-peptide 3R antibody recognizes the configuration of the D-β-Asp isomer in the T18 and T6SA peptides. Both T18 and T6 SA peptides have common sequence, namely Leu-Asp-Ala, and the result suggests that the antibody recognizes the LD(Dβ)A sequence of the antigen. As shown in Figure 5, the anti-peptide 3R antibody also did not react with the D-β-Asp isomer in the T6SR peptide (TVLDRGISEVR), in which the Ser is replaced by Arg. Figure 6 shows the reactivity of anti-peptide 3R antibody toward the T1 peptide of βB2-cystallin (MASD^4^HQTQAGK). The antibody did not recognize the D-β-Asp residue in the T1 peptide of human βB2-crystallin. This result is unsurprising because the T1 peptide of βB2-cystallin does not contain Leu or Ala in the neighborhood of the D-β-Asp residue.

Figure 7 shows the reactivity between anti-peptide 3R antibody and the isomers of T18LR of αΑ-cystallin (IQTGRDATHAER). The antibody distinguishes between the D-β-Asp residue and the L-α-Asp in the peptide. However, the intensity is weak compared with reactivity toward the D-β-Asp residue in T18 or T6 SA peptides. Furthermore, we examined whether the antibody recognizes the D-β-Asp residue in a peptide from keratin 10 (mouse) representing amino acids 330–341 (NRKDAEEWFNQK; Figure 8). The reactivity toward the D-β-Asp residue of this peptide was 20% that of T18 peptide. We are able to summarize the above results as follows: the antibody recognizes D-β-Asp-Ala and in addition, the antibody has strong affinity for peptides containing Leu-D-β-Asp-Ala. However, the antibody cannot recognize D-β-Asp-Y when Y is not Ala.

The existence of proteins containing a D-β-Asp residue in the sequence Asp-Ala, such as Asp^151^-Ala in human αA-crystallin and Asp^1^-Ala in β−amyloid protein is widely known. The Asp in these sites is regularly found to be inverted from L- to D-Asp under physiologic conditions. Therefore this antibody that recognizes D-β-Asp-Ala is very useful for detection of D-β-Asp containing protein in the living tissue. The inversion of Asp occurs via a succinimide intermediate as follows: (i) When the carbonyl group of the side chain of the L-α-Asp residue is attacked by the nitrogen of the amino acid residue following the Asp residue, L-succinimide is formed by intramolecular cyclization; (ii) L-succinimide may be converted to D-succinimide through an intermediate that has the prochiral α-carbon in the plane of the ring; (iii) The D- and L-succinimide are hydrolyzed at either side of their two carbonyl groups, yielding both β- and α-Asp residues, respectively. Thus, four isomers, L-α-Asp, L-β-Asp, D-α-Asp and D-β-Asp, are simultaneously formed in the protein. The rate of succinimide formation is expected to depend on the residue neighboring the Asp. When the neighboring amino acid of the Asp residue has a small side chain, as with glycine, alanine or serine, formation of the succinimide intermediate occurs easily because there is no steric hindrance. Moreover, when L-α-Asp inverts to D-Asp, about 70% of D-Asp is the β form, and thus the D-β-Asp isomer predominates over the other isomers. In fact, using this antibody, D-β-Asp-containing peptide/proteins were observed in nonpigmented ciliary epithelial cells, in drusen, and in the sclera [15] of eye and in sun-damaged skin of elderly donors [11]. However, the detection of D-β-Asp containing protein using this antibody is limited by the sequence surrounding the Asp residues. For example, although Asp 58 of αA-crystallin and Asp 4 of βB2-crystallin in the lens of elderly donors showed high D/L ratios, the anti-peptide 3R antibody does not react with these Asp residues because of the lack of an Asp-Ala sequence. These results indicate that experiments using only this antibody are insufficient for a thorough identification of D-Asp containing proteins. Instead, chemical analysis, including the purification of protein, the analysis of optical isomers of amino acids with HPLC is required. However, chemical analysis is very complicated and it takes a long time to obtain the D/L ratio of Asp in the protein. Therefore, as a first step in the detection of D-β-Asp-containing peptide/proteins in living tissues, immunohistochemical staining and western blotting using this antibody are useful. However, chemical analysis is still required after such a screening. The advantage of immunohistochemistry using this antibody is that it can show the localization of D-β-Asp-containing peptide/proteins in living tissues. However, to detect more D-β-Asp containing proteins, we need to synthesize the other 3R peptides, with different sequences for use as antigen. If antibodies against peptides containing 20 variations of Leu-D-β-Asp-X, (X is any amino acids) could be prepared, all D-β-Asp containing proteins could potentially be detected in living tissues.

Together with the chemical analysis, the anti-peptide 3R antibody is a powerful tool for the study of the mechanism of D-β-Asp formation and the study of aging of proteins and age-related diseases.
